# Phytochemical Analysis and Metabolic Profiling in Plants

**DOI:** 10.3390/plants15182754

**Published:** 2026-09-09

**Authors:** Yulian Voynikov

**Affiliations:** Department of Chemistry, Faculty of Pharmacy, Medical University of Sofia, 1000 Sofia, Bulgaria; y_voynikov@pharmfac.mu-sofia.bg

This Special Issue (SI) was launched to collect work applying UHPLC–HRMS and related techniques to plant secondary metabolites, together with clustering and multivariate methods, which are used to provide insights into relating MS data to structures. The SI published 10 articles covering the metabolomics of plant species from Europe and Asia, using HPLC–DAD, GC–MS, LC–ESI–MS, UHPLC–MS/MS, HPLC–ESI–ion trap MS/MS and Orbitrap HRMS.

## 1. Profiling of Underexplored Species

Babacan et al. identified 83 metabolites in *Eminium intortum* (Banks & Sol.) Kuntze and *E. spiculatum* (Blume) Schott by UHPLC–HRMS, the first such data for either species, and related them to antioxidant and enzyme inhibitory activity [1]. Dăescu et al. characterised 16 polyphenols in Romanian *Amelanchier lamarckii* F.G. Schroed., followed their bioaccessibility through simulated digestion, and screened the extract against tyrosinase, α-glucosidase and acetylcholinesterase [2]. Razgonova et al. combined supercritical CO_2_ extraction with HPLC–ESI–ion trap MS/MS to annotate 283 compounds in three Kamchatkan wild *Rosa* species (*R. acicularis* Lindl., *R. amblyotis* C.A. Mey. and *R. rugosa* Thunb.), with 48 of them being reported for the first time [3].

## 2. Variation Between Cultivars, Species and Populations

Niu et al. applied quasi-targeted metabonomics to four cultivars of *Mesona chinensis* Benth., annotating 1287 metabolites and attributing the higher antioxidant activity of the Xiaoye cultivar principally to flavonoids [4]. Chakarova et al. showed that the flavonoid profiles of *Cuscuta campestris* Yunck. and *C. epithymum* (L.) L. in Bulgaria separate the two species independently of locality or host plant, supporting their use as chemotaxonomic markers [5].

## 3. Growth Conditions and Postharvest Treatment

Perchuk et al. compared sprouts of mung bean, *Vigna radiata* (L.) R. Wilczek, germinated in darkness and in light, identifying more than 100 compounds by GC–MS and showing that saccharides, fatty acids and phenolics predominated in dark-grown material and amino acids in light-grown material [6]. Mahfuzur et al. applied widely targeted UPLC–MS/MS to SO_2_-treated longan (*Dimocarpus longan* Lour.) and attributed the characteristic sulfur flavour of the aril to increased glucosinolates and related amino acid derivatives [7].

## 4. Analytical Method Development

Akhtar et al. developed an HPLC–DAD protocol for nineteen polyphenols in fruits, vegetables and other plant foods [8]. Zhao et al. optimised UHPLC–Q-Exactive Orbitrap/MS conditions for proanthocyanidin monomers in persimmon (*Diospyros kaki* Thunb.) [9].

## 5. Data Analysis and Compound Annotation

Park et al. trained a vision transformer on ESI–MS/MS spectra and classified flavonoid glycoside isomers differing in substitution position with over 80% accuracy, independently of the adduct measured [10].
